# Suppressed OGT expression inhibits cell proliferation while inducing cell apoptosis in bladder cancer

**DOI:** 10.1186/s12885-018-5033-y

**Published:** 2018-11-20

**Authors:** Longsheng Wang, Shaojun Chen, Ziwei Zhang, Junfeng Zhang, Shiyu Mao, Jiayi Zheng, Yang Xuan, Mengnan Liu, Keke Cai, Wentao Zhang, Yadong Guo, Wei Zhai, Xudong Yao

**Affiliations:** 1 Department of Urology, Shanghai Tenth People’s Hospital, Tongji University, School of Medicine, Shanghai, 200072 China; 2 Department of pathology, Shanghai Tenth People’s Hospital, Tongji University, School of Medicine, Shanghai, 200072 China; 3 Shanghai Key Laboratory of Regulatory Biology, Institute of Biomedical Sciences, School of Life Sciences, East China Normal University, Shanghai, 200241 China

**Keywords:** Autophagy, Bladder cancer, DNA damage response, *O*-GlcNAcylation

## Abstract

**Background:**

This study aimed to explore hyper-*O*-linked *N*-acetylglucosaminylation (*O*-GlcNAcylation) with an elevation of the expression of *O*-linked-β-*N*-acetylglucosamine transferase (OGT) in human bladder cancer.

**Methods:**

Immunohistochemical staining for OGT and O-GlcNAcylation was performed in 20 paired human bladder cancer and adjacent normal tissues, as well as in human bladder cancer tissue microarrays (*N* = 169). The expression level of OGT and *O*-GlcNAcylation in cell lines were detected using the Western blot analysis. The effects of *O*-GlcNAcylation on the cell proliferation of bladder cancer were detected using 3-(4,5-dimethylthiazol-2-yl)-2,5-diphenyltetrazolium bromide (MTT) and clone formation assays. Cell apoptosis and cell cycle analysis were detected using flow cytometry. The autophagy of bladder cancer cells was investigated using the Western blot analysis, and GFP–LC3 plasmid was used to detect the autophagic flux. MTT assay was performed to detect the sensitivity of bladder cancer cells to cisplatin after OGT knockdown.

**Results:**

The expression of OGT and the *O*-GlcNAcylation were upregulated in bladder cancer tissues and cell lines. *O*-GlcNAcylation and OGT were observed in nucleus and cytoplasm and found to be higher in muscle-invasive bladder cancer (MIBC) than in non-muscle-invasive bladder cancer (NMIBC). Reducing hyper*-O*-GlcNAcylation by OGT knockdown inhibited the proliferation of bladder cancer cells in vitro and xenograft tumor growth in vivo, triggered apoptosis, as well as led to cell cycle arrest. It also increased autophagy in bladder cancer cells. This study demonstrated increased autophagy pro-survival, but not pro-death. Reducing hyper*-O*-GlcNAcylation by OGT knockdown facilitated the chemosensitivity of bladder cancer cells to *cis*-platinum.

**Conclusions:**

The data indicated that hyper-*O*-GlcNAcylation enhanced oncogenic phenotypes and was involved in DNA damage response in bladder cancer.

## Background

Bladder cancer is a common malignant tumor with high mortality. The morbidity of bladder cancer has steadily increased during these years [1]. A total of 60,490 new diagnosed bladder cancer patients in the United States, and about 12,240 of these patients died of the disease in 2017 [2]. Besides traditional surgical treatment, targeted chemotherapy and immunotherapy have gained importance in treating bladder cancer [3–6]. However, 25% of bladder cancer is reported to be muscle invasive, and approximately 50% of these patients die from a life-threatening metastasis within 2 years [6, 7]. Generally, bladder cancer is characterized by hematuria, dysuria, and urination. However, few specific biomarkers of bladder cancer have been identified to date.

Overnutrition with modern lifestyle is a crucial risk factor for cancer, and *O*-GlcNAcylation reflects the glucose status of cells [8]. About 2–3% of glucose entering the cell passes through the hexosamine biosynthetic pathway (HBP), which regulates the *O*-GlcNAcylation of intracellular proteins [9]. Analogous to phosphorylation, *O*-linked *N*-acetylglucosaminylation (*O*-GlcNAcylation) is a invertible post-translational modification that influences almost all cellular processes [9–11]. OGT is critical for *O*-GlcNAcylation, it adds the *O*-GlcNAc moiety to the free hydroxyl group of selected serine and threonine residues on proteins, which is removed by *O*-GlcNAc-selective *N*-acetyl-β-D-glucosaminidase (*O*-GlcNAcase, OGA) [12]. *O*-GlcNAcylation has been reported to modulate cell functions, and multiple proteins have been demonstrated with O-GlcNAc modification over these years [11]. The deregulation of *O*-GlcNAcylation has been implicated in various diseases [13, 14]. Moreover, various studies indicated that *O*-GlcNAcylation was upregulated in various cancers and might be related to various hallmarks of cancer, such as cell proliferation, survival, metastasis, invasion, and so forth [15]. Rozanski et al. reported mRNA expression of OGT in the urine of 51.7% patients with bladder cancer, but not in the urine of healthy individuals [16]. However, no further studies about OGT and *O*-GlcNAcylation in bladder cancer have been reported to date.

Autophagy is a cellular defensive pathway under unfavorable circumstances. It is imperative to cell survival [17]. However, autophagy induces cell death under certain conditions [18, 19]. Extensive evidence is available on the role of autophagy in the progression of various diseases such as cancer [20, 21]. Autophagy may prevent tumor progression and improve the efficacy of cancer therapy [22]. Increased *O*-GlcNAcylation has been reported to inhibit autophagy [23]. In addition, the inhibition of *O*-GlcNAcylation seems to facilitate autophagosome formation and increase autophagic flux [23, 24].

Surgery combined with chemotherapy and/or radiotherapy is the current strategy for treating bladder cancer. Cisplatin-based chemotherapy is the mainstay of both muscle-invasive and metastatic bladder cancer [25]. Cisplatin causes DNA damage, leading to apoptosis and cell death [26]. OGT was found to mediate histone *O*-GlcNAcylation, regulating DNA damage response (DDR) [27]. Moreover, Miura et al. demonstrated that O-GlcNAc modification affected the ATM-mediated DDR. Whether DDR is regulated by *O*-GlcNAcylation in bladder cancer needs to be further explored.

This study aimed to demonstrate the expression and function of OGT and O-GlcNAc modification in bladder cancer. The results advanced the understanding of the tumor-promoting effect of OGT and *O*-GlcNAcylation in bladder cancer.

## Methods

### Materials and reagents

Anti-OGT antibody was obtained from Bioworld Technology, Co. (Nanjing, China). Other primary antibodies were purchased from Abcam (Cambridge, MA, USA). Fluorescent-labeled secondary antibodies were procured from Jackson Immuno Research. Cisplatin was obtained from Selleckchem (TX, USA). Autophagy inhibitor chloroquine (CQ) was obtained from Sigma–Aldrich (MO, USA).

### Tumor microarray and immunohistochemical analysis

The paraffin sections were taken from 169 patients with bladder cancer (85 patients with NMIBC and 84 with MIBC) for tissue microarray. All these samples were obtained from the Department of Urology, Shanghai Tenth People’s Hospital, Tongji University (Shanghai, China) from 2013 to 2016. Tumor-rich areas were board-certified by the pathologist. After constructing the tissue microarray, the samples were stained for *O*-GlcNAcylation (RL2) and OGT. The intensity of the staining was scored using the H-score method (3D HISTECH, H-SCORE = ∑(PI × I) = (percentage of cells of weak intensity × 1) + (percentage of cells of moderate intensity × 2) + (percentage of cells of strong intensity × 3) [28].

### Cell culture and transient transfection

Human bladder epithelial permanent cell (sv-huc-1) and human bladder cancer cell lines (T24, UMUC-3, 5637 and EJ) were obtained from the Institute of Cell Research of the Chinese Academy of Sciences (Shanghai, China). Cells were cultured in Roswell Park Memorial Institute 1640 media/Dulbecco’s Modified Eagle Medium/F12 K (Invitrogen, Carlsbad, CA, USA) added with 10% fetal bovine serum (FBS) at 37 °C in 5% CO_2_. Lipofectamine 2000 (Invitrogen, CA, USA) was used for transfection in accordance with the manufacturer’s protocol. si-OGT and si-NC were all obtained from GenePharma (Shanghai, China). The sequence was 5’-GAAGAAAGUUCGUGGCAAA-3′ for si-OGT. The transfection efficiency was detected using Western blot analysis after cultivation for 48 h.

### Western blot analysis

Total protein of cells or tissues was extracted using precooled radio-immunoprecipitation assay lysis buffer with protease inhibitor. An equal amount of protein was separated using 10% sodium dodecyl sulfate–polyacrylamide gel electrophoresis and then transferred onto nitrocellulose membranes. Afterward, the membranes were incubated overnight with specific primary antibodies at 4 °C. Then, the membranes were incubation with secondary antibodies for 2 h. The signals were identified by electro chemiluminescent detection.

### Cell proliferation in vitro

Cell proliferation was measured using the MTT assay as previously described [29]. The lentivirus expressing shRNAs against OGT was produced by Jiman Co. (Shanghai, China). UMUC-3 cells were infected with LV-sh-OGT or LV-sh-NC and then selected using puromycin (Sigma–Aldrich). The expression of OGT and *O*-GlcNAc was examined at RNA and protein levels. For colony formation assays, LV-sh-OGT or LV-sh-NC cells were plated in six-well plates with a density of 1 × 10^3^/well. After cultivation for 10 days, the plates were methanol-fixed, and then stained with 0.1% crystal violet.

### Xenograft assays in nude mice

After generating stably transfected LV-sh-NC and LV-sh-OGT cell lines, the cells (3 × 10^6^ in 0.2 mL of PBS) were implanted subcutaneously into the dorsal flanking sites of male BALB/c nude mice (*N* = 10 in each group, 6 weeks). The tumorigenic potential was evaluated 3 weeks after inoculation. The mice were sacrificed using pentobarbital overdose (1%), and tumors were weighed and excised. The animal care and experiments were carried out under the National Institutes of Health Guide for Care and Use of Laboratory Animals. All animal studies were approved by the Institutional Animal Care and Use Committee of the Shanghai Tenth People’s Hospital of Tongji University.

### Flow cytometry

For the apoptosis assay, Annexin V–fluorescein isothiocyanate (FITC) detection kit (BD Biosciences, Erembodegem, Belgium) was used according to the manufacturer’s introduction. The cells were collected, washed twice with PBS, resuspended in Annexin V–FITC and propidium iodide (PI), and stained in the dark for 15 min at room temperature [29]. Subsequently, the cell apoptosis rate was analyzed using flow cytometry (fluorescence-activated cell sorting, BD Biosciences). For cell cycle distribution analysis, the cells were harvested and fixed in 70% ice-cold ethanol overnight. Then, the cells were centrifuged and resuspended in PBS containing PI (BD Biosciences) and RNase (100 μg/mL) as well as Triton X-100 (0.2%) for 30 min. Finally, flow cytometry was used to analyze cell cycle distribution [29]. The tests were performed three times for each sample.

### GFP-LC3 puncta assay

The GFP-LC3 plasmid was used in this study to monitor autophagy. In brief, the cells were cultured and transfected with GFP-LC3 plasmid for 24 h. Then, they were treated, cell images were chosen randomly under a confocal microscope, and the number of puncta was calculated.

### Statistical analysis

The statistical analysis was performed using GraphPad Prism 5 (GraphPad Prism Software Inc., CA, USA) and SPSS 23 (SPSS, Inc., IL, USA). The data were presented as mean ± standard deviation (SD). The differences between two groups were analyzed using the Mann–Whitney *U* test or two-tailed unpaired Student *t* test. In all analyses of this study, a *P* value < 0.05 was considered statistically significant.

## Results

### *O*-GlcNAcylation and the expression of OGT were upregulated in bladder cancer cell lines and tissue specimens

**Fig. 1:**
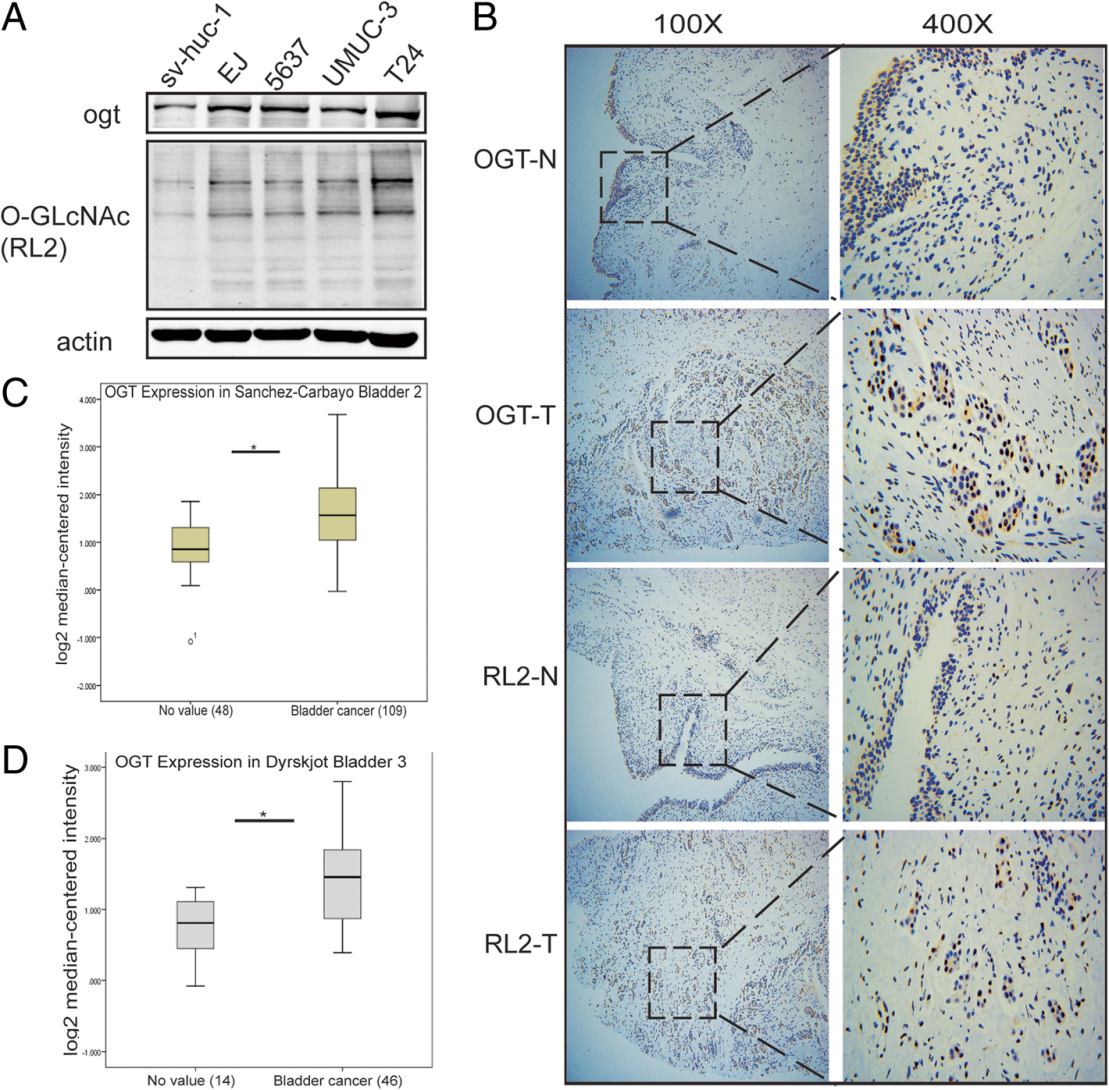
*O*-GlcNAcylation and the expression of OGT were upregulated in bladder cancer cell lines and tissue specimens. (**a**) *O*-GlcNAcylation and expression of OGT in bladder cancer cell lines (EJ, 5637, T24, and UMUC-3) and human bladder epithelial permanent cell (sv-huc-1) line. (**b**) *O*-GlcNAcylation and expression of OGT in bladder cancer tissues and adjacent normal tissues. (**c** and **d**) Oncomine expression analysis of OGT in human bladder cancers and normal bladder tissues

Previous studies revealed that *O*-GlcNAcylation was upregulated in various cancers [30–35]. Therefore, the present study explored whether it was elevated in bladder cancer. First, *O*-GlcNAc modification levels were examined in human bladder cancer cell lines compared with human bladder epithelial permanent cell line (sv-huc-1). The result suggested that *O*-GlcNAcylation and expression of OGT increased in bladder cancer cells (Fig. 1a). Consistently, the immunohistochemical (IHC) analysis illustrated an obvious augmentation of *O*-GlcNAcylation and expression of OGT in clinical tumor samples of bladder than in adjacent normal tissues (Fig. 1b). In addition, *O*-GlcNAcylation and expression of OGT were observed in both nucleus and cytoplasm. Interestingly, the Oncomine expression analysis based on previous studies also demonstrated that the expression of OGT was higher in bladder cancer tissues than in normal tissues, suggesting a higher level of *O*-GlcNAc modification in bladder cancer tissues (Fig. 1c and d).

**Fig. 2:**
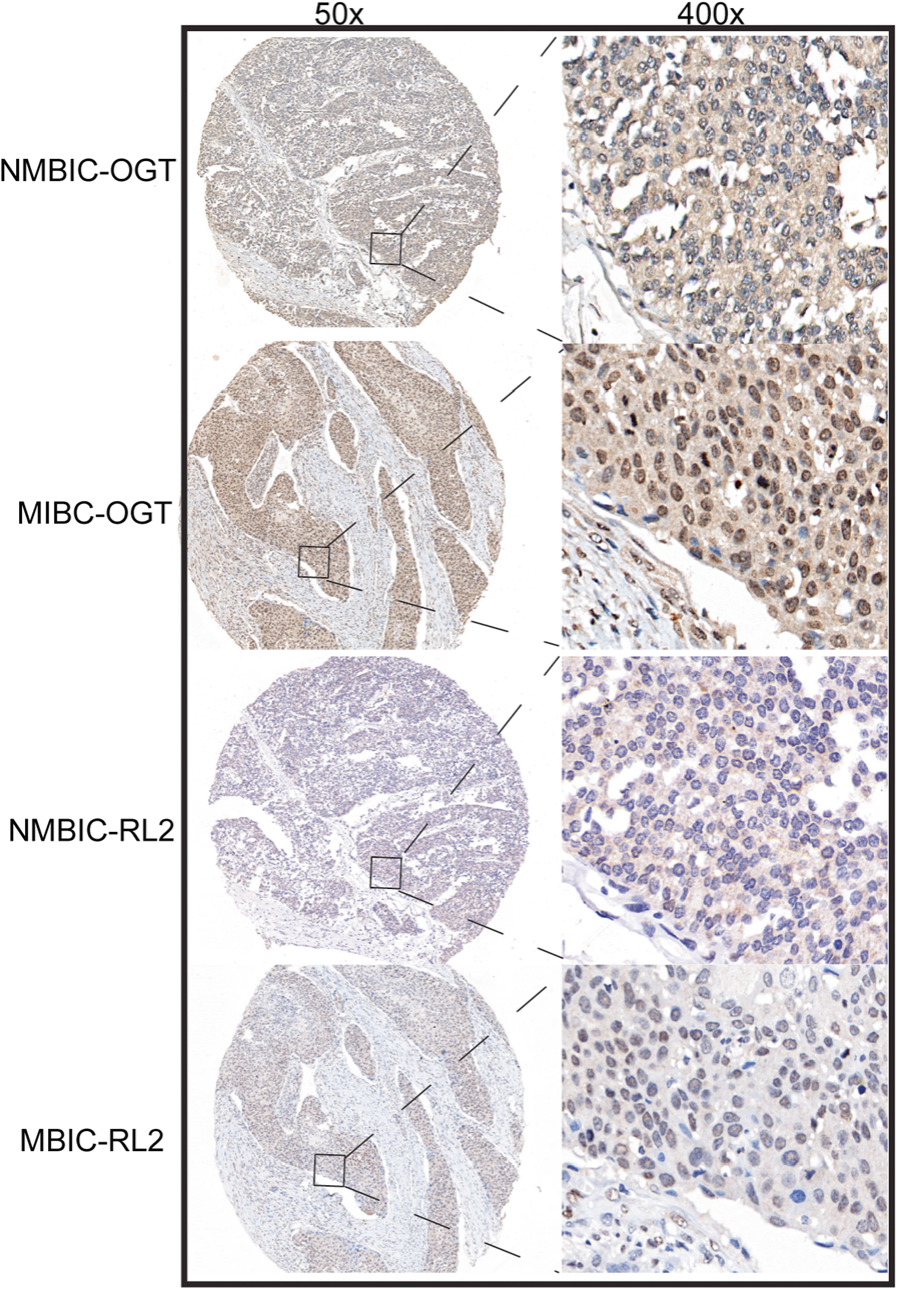
*O*-GlcNAcylation and expression of OGT in NMIBC and MIBC tissues. Representative images of *O*-GlcNAcylation and expression of OGT in MIBC and NMIBC

Next, a total of 85 tumor tissue samples of NMIBC and 84 samples of MIBC were prepared for the tissue microarrays (TMA). The IHC analysis was carried out for *O*-GlcNAcylation (RL2) and OGT, and the stained tissue microarrays were scanned using Panoramic MIDI (3D HISTECH). *O*-GLcNAcylation and expression of OGT were analyzed using the H-score method. As shown in Fig. 2, *O*-GlcNAcylation and expression of OGT in both NMIBC and MIBC tissues were localized in the cytoplasm and nucleus. In overall tissues, *O*-GlcNAcylation and expression of OGT were positively correlated with each other (*r* = 0.347, *P* < 0.001 by Pearson correlation coefficient). Table 1 shows *O*-GlcNAcylation and expression of OGT according to clinicopathological features. *O*-GLcNAcylation and expression of OGT were found to be effectively higher in MIBC than in NMIBC tissues. A relatively higher *O*-GlcNAcylation and expression of OGT in the nucleus was found in MIBC. Nevertheless, a higher cytoplasmic expression was observed in NMIBC (Fig. 2). Furthermore, *O*-GlcNAcylation and expression of OGT were found to be higher in patients with a higher BMI.

**Table 1:** O-GlcNAcylation and expression of OGT according to clinicopathological parameters in bladder cancer (*P < 0.05; **P < 0.01)

Characteristic		O-GlcNAcylation score	OGT score	P value of O-GlcNAcylation	P value of OGT
Gender	male(126)	68.12 ± 44.01	107.32 ± 37.57	0.258	0.576
	female(43)	63.32 ± 38.14	103.32 ± 40.47		
Age (y)				0.379	0.608
	< 65(84)	64.14 ± 41.99	108.77 ± 34.26		
	≥65(85)	69.38 ± 43.10	104.09 ± 41.58		
Body mass index (kg/m2)				0.022*	0.001**
	< 24.7(84)	59.19 ± 42.12	96.20 ± 41.00		
	≥.001(85)	74.52 ± 41.79	116.29 ± 32.59		
T stage				< 0.01**	0.005*
	NMIBC(85)	50.18 ± 37.37	99.51 ± 36.53		
	MIBC(84)	83.82 ± 40.89	113.18 ± 38.93		
Lymph node metastasis				0.630	0.019
	NO(83)	68.04 ± 42.17	112.60 ± 36.48		
	YES(86)	65.80 ± 43.10	100.23 ± 39.13		
pathology grade				0.742	0.335
	LOW(59)	68.26 ± 45.69	100.32 ± 44.24		
	HIGH(110)	66.17 ± 40.93	109.52 ± 34.41		
M stage				0.826	0.243
	NO(80)	66.19 ± 40.52	102.38 ± 38.65		
	YES(89)	67.54 ± 44.48	109.84 ± 37.75		

### Reduction of OGT inhibited the growth of bladder cancer cells in vitro and xenograft tumor growth in vivo

**Fig. 3:**
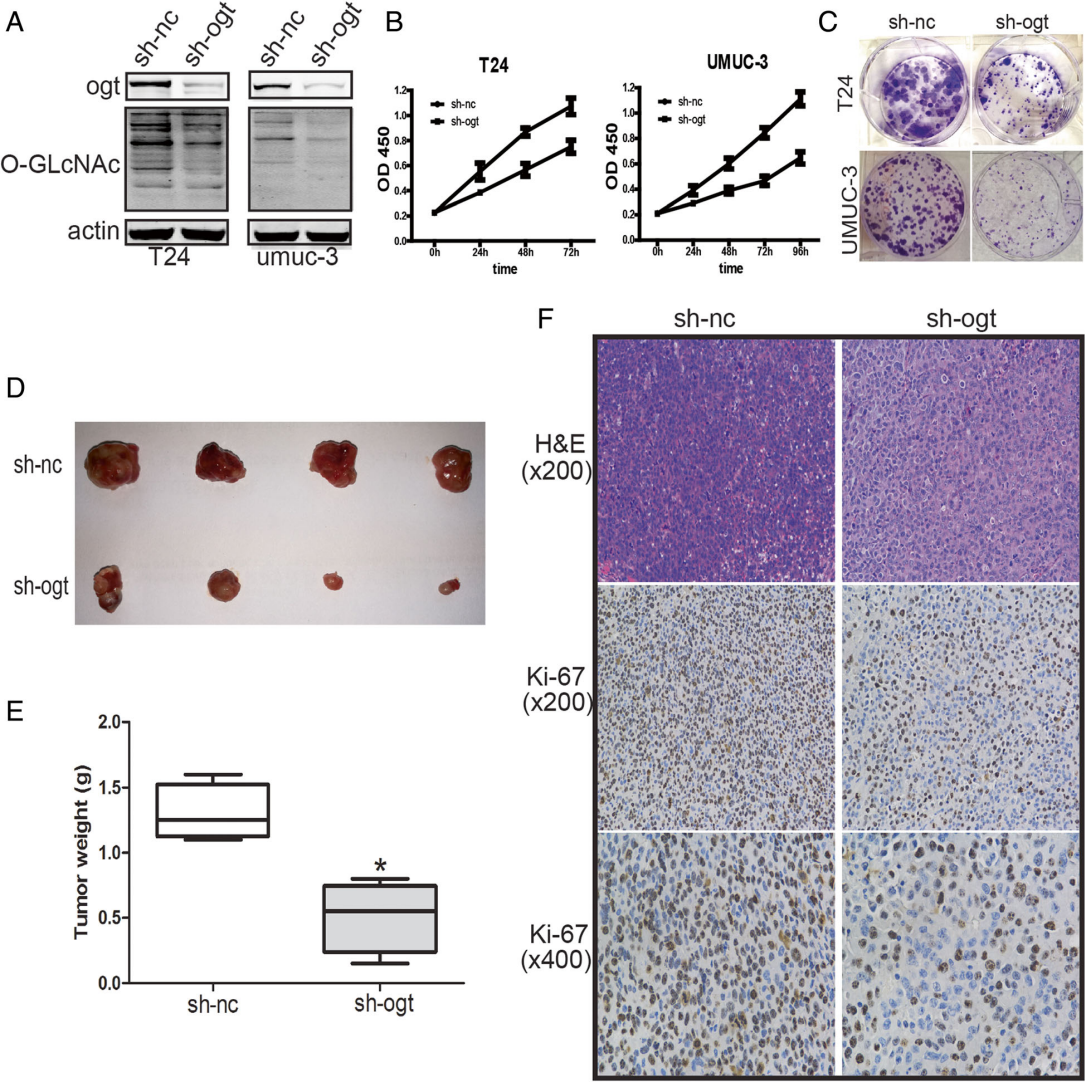
Reduction of OGT inhibited the growth of bladder cancer cells in vitro and xenograft tumor growth in vivo*.* (**a**) The expression of OGT and *O*-GlcNAcylation after infection with LV-sh-OGT or LV-sh-NC. MTT assay (**b**) and cell colony formation assay (**c**) were used to determine the proliferation of T24 and UMUC-3 cells after transfection with LV-sh-OGT and LV-sh-NC. Following infection with LV-sh-OGT or LV-sh-NC, UMUC-3 cells were implanted subcutaneously in 6-week-old nude mice. The tumor growth was evaluated following 3 weeks of tumor implantation. Representative images (**d**) and weight (**e**) of the excised tumors derived from nude mice are shown. (**f**) Representative images of hematoxylin and eosin staining and Ki-67 immunohistochemical detection of the excised tumors derived from nude mice. Data are shown as mean ± standard deviation of three independent experiments. *P < 0.05

It is well established that *O*-GlcNAcylation is vital for the proliferation of various cancer cells. Further, downregulating OGT inhibits tumor formation and growth [9, 31, 36]. *O*-GLcNAcylation in T24 and UMUC-3 cells was downregulated by OGT knockdown to determine whether *O*-GlcNAcylation affected the proliferation of bladder cancer cells (Fig. 3a). Subsequently, the results of MTT assay indicated that the OGT knockdown–mediated reduction of *O*-GlcNAcylation potently inhibited the growth of bladder cancer cells (Fig. 3b). Moreover, the colony formation assay indicated the proliferation of T24 and UMUC-3 cells was suppressed dramatically following infection with LV-sh-OGT (Fig. 3c).

Moreover, the xenograft tumor growth assay was performed after establishing stable LV-sh-OGT UMUC-3 cells. The mice bearing tumors were sacrificed after 3 weeks of tumor implantation. Then, the expression of Ki-67 was examined immunohistologically. A remarkable reduction in the size and weight of tumors was found in the LV-sh-OGT group than in the LV-sh-NC group (Fig. 3d and e). As shown in Fig. 3f, the Ki-67 levels was lower in tumor tissues from the OGT knockdown group than in the control group, indicating a tumor-suppressing potential.

### Reduction of OGT promoted bladder cancer cell apoptosis and inhibited cell cycle distribution

**Fig. 4:**
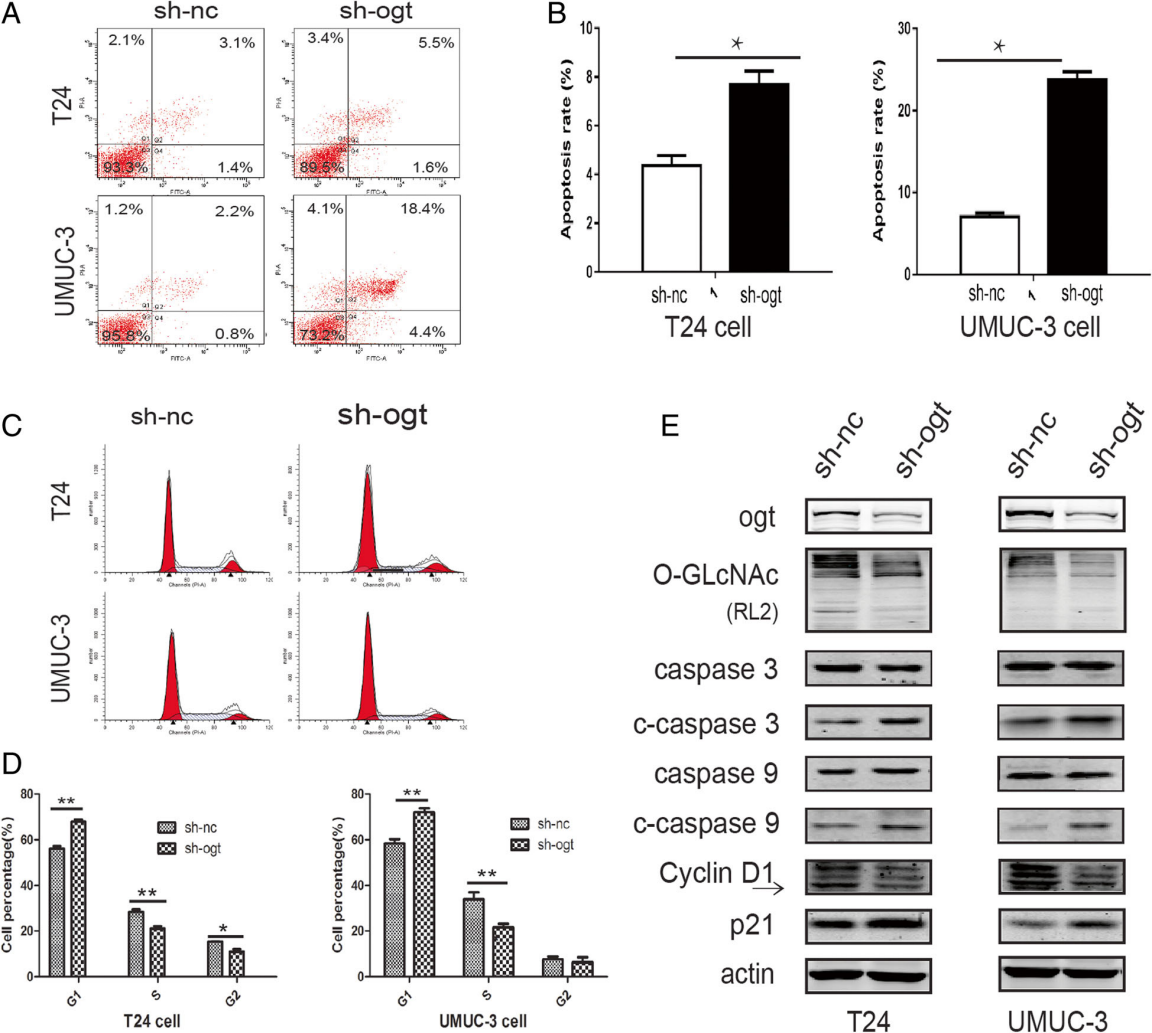
Reduction of OGT promoted bladder cancer cell apoptosis and inhibited cell cycle distribution. (**a** and **b**) Apoptosis of T24 and UMUC-3 cells was measured by flow cytometry following OGT knockdown. (**c** and **d**) Effects of OGT knockdown on cell cycle distribution of T24 and UMUC-3 cells determined using flow cytometry. (**e**) Representative cell apoptosis and cell cycle markers were examined using the Western blot analysis. Data are presented as mean ± standard deviation of three independent experiments. *P < 0.05

The flow cytometry was used to detect the effects of OGT on bladder cancer cell apoptosis and cell cycle distribution. The results showed that the apoptosis of T24 and UMUC-3 cells were enhanced after the downregulation of OGT following infection with LV-sh-OGT compared with LV-sh-NC (Fig. 4a and b). The expression of cleaved caspase-3 and cleaved caspase-9, having a pivotal role in the apoptotic process, was found to be upregulated in OGT knockdown cells (Fig. 4e). In addition, the cell cycle analysis confirmed that G0/G1 cell population increased with a decrease in cells in the S and G2/M phases in LV-sh-OGT bladder cancer cells compared with control groups (Fig. 4c and d). Cyclin D1 was reported to be closely involved in cell cycle distribution. The p21 protein is a well-known cyclin-dependent kinase (CDK) inhibitor that participates in cell cycle regulation via inhibiting cyclin–CDK complex activity in the G1 phase [29]. Thus, the expression of cyclin D1 and p21 was examined. The OGT knockdown–induced downregulation of *O*-GlcNAcylation was found to obviously upregulate the expression of p21 and downregulate cyclin D1 (Fig. 4e).

Taken together, the data indicated that OGT knockdown attenuated the proliferation of bladder cancer cells probably due to apoptosis promotion and cell cycle inhibition.

### Downregulation of OGT effectively induced autophagy, which had a pro-survival role in human bladder cancer cells

**Fig. 5:**
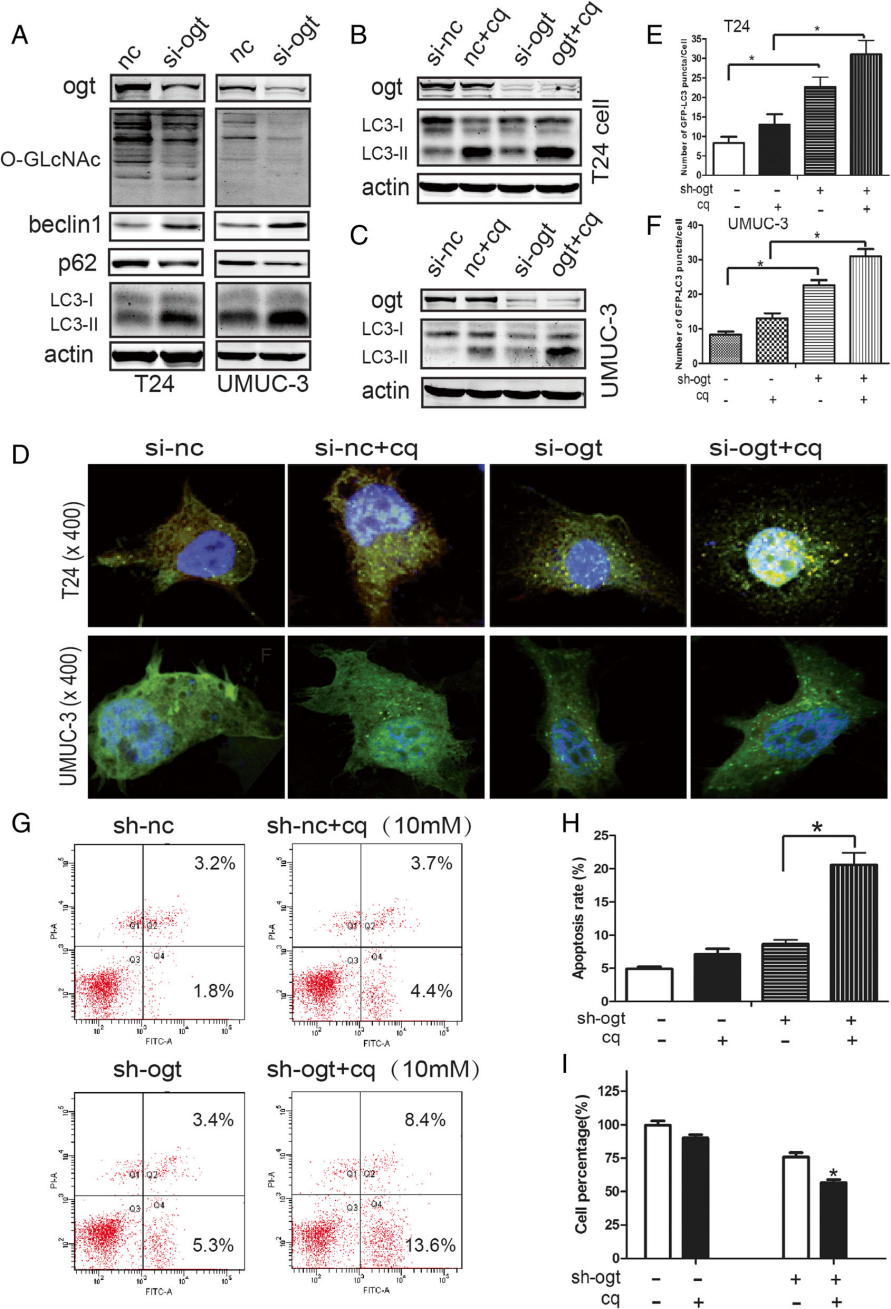
Downregulation of OGT effectively induced autophagy, which had a pro-survival role in human bladder cancer cells. (**a**) Expression of autophagy markers (LC3, P62, and Beclin 1) was examined using the Western blot analysis. T24 (**b**) and UMUC3 (**c**) cells were transfected with si-OGT, and with or without 10 μM CQ, for 24 h, and the protein levels of LC3 were assessed using the Western blot analysis. Transfected T24 and UMUC3 cells were transfected with the *GFP-LC3* construct and the transfectants were treated with or without CQ (10 μM) for 24 h. (**d**) Representative images of GFP-LC3 puncta were captured using a confocal fluorescence microscope (magnification, × 400). (**e** and **f**) Number of puncta per GFP-LC3-positive cell was calculated and presented. (**g** and **h**) T24 cells were transfected with si-OGT or si-NC, and with or without 10 μM CQ for 24 h. Apoptosis was measured using flow cytometry, and cell viability was examined using MTT assay (I)

In light of several reports indicating that *O*-GlcNAc modification is essential for regulating autophagy [23, 37], the potential interplay between autophagy and OGT knockdown–mediated antitumor effects was further investigated. In this study, *O*-GlcNAcylation in T24 and UMUC3 cells was downregulated with si-OGT. As shown in Fig. 5a, the downregulation of OGT led to a salient increase in the LC3 conversion from LC3-I to LC3-II, which is a hallmark of autophagy. Autophagic flux was detected by examining the expression of p62, which was degraded in autolysosomes. As shown in Fig. 5a, p62 expression decreased in the si-OGT group, suggesting an increase in autophagic flux. Moreover, the expression of Beclin 1, which was required for initiating autophagic vesicle formation, was upregulated in the OGT knockdown group compared with the NC group (Fig. 5a). In brief, the results indicated that the si-OGT-induced downregulation of *O*-GlcNAcylation effectively drove autophagy in human bladder cancer cells.

Furthermore, stable T24 and UMUC-3 cells transfected with GFP-LC3 plasmid were established to further demonstrate autophagy induction by the si-OGT-induced downregulation of *O*-GlcNAcylation. The autophagy inhibitor chloroquine (CQ, 10 μm) was used to further assess autophagy. The si-OGT-induced downregulation of *O*-GlcNAcylation led to the further accumulation of LC3-II (Fig. 5b and c) and GFP-LC3 puncta (Fig. 5d–f) in the presence of CQ.

The interplay between apoptosis and autophagy varies in regulating cell survival and death between cell types and different stresses [22]. OGT downregulation–induced apoptosis in T24 cells further increased with CQ treatment, blocking autophagosome–lysosome fusion (Fig. 5g and h). The MTT assay was performed to examine cell viability after treatment with CQ so as to determine the role of autophagy in the reduction of OGT. The viability of T24 cells was found to be restored in the sh-OGT group (Fig. 5g and h). These results indicated that OGT downregulation–induced autophagy had a pro-survival role in bladder cancer cells.

### Downregulation of OGT increased the sensitivity of bladder cancer cells to cisplatin

**Fig. 6:**
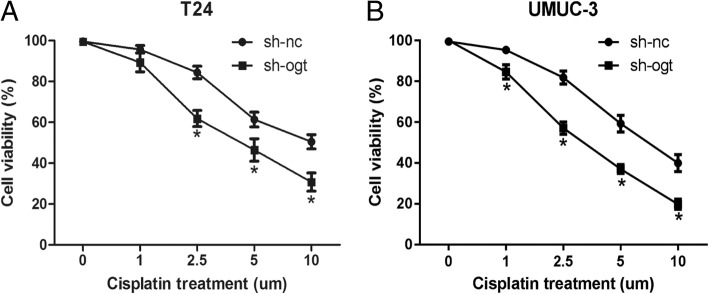
Downregulation of OGT increased the sensitivity of bladder cancer cells to cisplatin. T24 (**a**) and UMUC-3 (**b**) cell were transfected with LV-sh-OGT or LV-sh-NC, and then treated with various concentrations (0, 1, 2.5, 5, and 10 μM) of cisplatin. The cell viability was detected at various time points

*O*-GlcNAcylation was reported to be related to DDR [27, 38]. Therefore, the MTT assay was performed to detect the effects of OGT on the sensitivity of bladder cancer cells to cisplatin. Bladder cancer T24 and UMUC-3 cells were transfected with LV-sh-OGT or LV-sh-NC and then treated with various concentrations of cisplatin for 48 h. As shown in Fig. 6, the IC_50_ value of cells transfected with sh-OGT decreased markedly compared with that of the control cells (T24 cells, 4.64 μm vs 8.64 μm; UMUC-3 cells, 3.381 μm vs 7.04 μm). The result suggested that the reduction of OGT could elevate the sensitivity of bladder cancer cells to cisplatin.

## Discussion

Nutritional conditions can regulate tumor development by affecting the signaling pathways involved in tumor cell growth [9, 39]. Increased glucose intake in cancer cells contributes to increased HBP flux. Thus, *O*-GlcNAcylation levels rise in response to elevated UDP-GlcNAc, as the product of HBP flux. Recent studies reported that increased *O*-GlcNAcylation is a common feature of various tumors and contributes to transformed phenotypes [9, 10, 15]. Hyper-*O*-GlcNAcylation has been reported to be correlated with the excessive growth of cancer cells by regulating key proteins that modulate cell cycle progression [40]. In addition, hyper-*O*-GlcNAcylation was verified to have an anti-apoptotic influence in cancer cells. Moreover, previous studies also showed that hyper-*O*-GlcNAcylation was associated with cancer cell invasion, metastasis, and angiogenesis [30, 32, 33]. Therefore, it is believed that the suppression of hyper-*O*-GlcNAcylation may be a therapeutic target for various types of cancers.

A previous study demonstrated that a high mRNA level of OGT was associated with poor differentiation of bladder cancer cells [16]. However, further studies about *O*-GlcNAcylation in bladder cancer are lacking. The *O*-GlcNAcylation level in cell lines and clinical tissues was examined to explore the potential role of *O*-GlcNAcylation in bladder cancer. The present study testified that hyper-*O*-GlcNAcylation was associated with the upregulated OGT level in bladder cancer cells. Meanwhile, it was demonstrated that the *O*-GlcNAcylation level was higher in clinical bladder cancer tissues than in normal bladder tissues. Notably, the *O*-GlcNAcylation level was higher in MIBC tissues than in NMIBC tissues. Hyper-*O*-GlcNAcylation and overexpression of OGT have been described in various cancer types, including lung, breast, colon, liver, prostate, and endometrial [30, 32, 34, 35, 41, 42]. Therefore, *O*-GlcNAcylation has been suggested as a new cancer hallmark.

The hyper-*O*-GlcNAcylation of bladder cancer cells was reduced by OGT knockdown and its effects on phenotypes were examined. The OGT knockdown–induced reduction of hyper-*O*-GlcNAcylation suppressed the proliferation of bladder cancer cells in vitro and subcutaneous xenograft tumor growth in nude mice. The present study reported that OGT knockdown–induced cell proliferation inhibition might be due to apoptosis increasing and cell cycle arrest. These data suggested that the inhibition of OGT might be a potential therapeutic strategy in bladder cancer.

Hyper-*O*-GlcNAcylation has been suggested to blunt autophagy [23]. The present study reported that the reduction of *O*-GlcNAcylation by OGT knockdown potently increased autophagic flux in T24 and UMUC-3 cells. Autophagy had dual roles in cancer modulation. The study demonstrated the pro-survival role of *O*-GlcNAcylation reduction–induced autophagy in bladder cancer cells. Further experiments are needed to explore the mechanism underlying this phenotype.

The interaction between apoptosis and autophagy elicited physiopathological changes in different cell types and stresses. Chen et al. reported that autophagy suppression enhanced PT-induced apoptosis and cell death in bladder cancer cells [22], indicating a pro-survival role of interdependence between apoptosis and autophagy. On the contrary, the inhibition of autophagy attenuated FTY720-induced apoptosis [43]. The present study also revealed that the downregulation of *O*-GlcNAcylation by OGT knockdown–induced apoptosis and autophagy (Fig. 4a and b, and Fig. 5g and h). Cell apoptosis increased during the inhibition of autophagy, and cell viability was not restored after autophagy inhibition (Fig. 5i). These results suggested that autophagy exerted a tumor-promoting effect during the downregulation of *O*-GlcNAcylation in bladder cancer cells.

*O*-GLcNAcylation was reported to be involved in regulating protein function, stability, or localization during post-translational modification. DNA damage induced by chemicals or x-rays led to changes in the *O*-GlcNAcylation of cellular proteins. A previous study proved that *O*-GlcNAcylation affected DDR by modulating key proteins involved in DDR [27, 38]. The project of cisplatin/gemcitabine (GC project) is the first-line therapy for locally advanced or metastatic bladder cancer [44]. This study demonstrated that OGT knockdown pronouncedly increased the chemosensitivity of bladder cancer cells to cisplatin. This finding indicated that hyper-*O*-GlcNAcylation in bladder cancer might promote chemoresistance of cells to DNA-damaging agents, and the chemosensitivity might be rescued by reducing hyper-*O*-GlcNAcylation. Although *O*-GlcNAcylation might be involved in DDR, the details of this involvement need further investigation.

In summary, this novel study reported the role of *O*-GlcNAcylation in bladder cancer. It elucidated hyper-*O*-GlcNAcylation and deregulated expression of OGT in bladder cancer cells and clinical samples. It also demonstrated that cell proliferation was inhibited by the OGT knockdown–induced downregulation of *O*-GlcNAcylation in vitro and in vivo. In addition, autophagic flux was increased by OGT knockdown, and autophagy had a pro-survival role. The study further showed that the chemosensitivity of cells to DNA-damaging agent cisplatin was increased by OGT knockdown.

A limitation of the present study was that the mechanism underlying the biological effects of OGT and O-GlcNAcylation in bladder cancer was not explored. Future studies should aim to investigate the potential mechanism.

## Conclusion

Taken together, the findings indicated that the analysis of *O*-GlcNAcylation or expression of OGT might be useful in bladder cancer diagnostics, and OGT might be used as a potential target for bladder cancer therapy in the future.

## Abbreviations

BMI: Body mass index
CQ: Chloroquine
DDR: DNA damage response
HBP: Hexosamine biosynthetic pathway
MIBC: Muscle-invasive bladder cancer
NMIBC: Non- muscle-invasive bladder cancer
OGA: *O*-GlcNAc-selective *N*-acetyl-β-D-glucosaminidase (*O*-GlcNAcase)
*O*-GlcNAc: *O*-linked beta-*N*-acetylglucosamine
OGT: *O*-linked-β-*N*-acetylglucosamine transferase
TMA: Tissue microarrays
